# Optical vector analysis with attometer resolution, 90-dB dynamic range and THz bandwidth

**DOI:** 10.1038/s41467-019-13129-x

**Published:** 2019-11-13

**Authors:** Ting Qing, Shupeng Li, Zhenzhou Tang, Bindong Gao, Shilong Pan

**Affiliations:** Key Laboratory of Radar Imaging and Microwave Photonics, Ministry of Education, Nanjing University of Aeronautics and Astronautics, Nanjing, 210016 China

**Keywords:** Microwave photonics, Optical metrology

## Abstract

Optical vector analysis (OVA) capable of achieving magnitude and phase responses is essential for the fabrication and application of emerging optical devices. Conventional OVA often has to make compromises among resolution, dynamic range, and bandwidth. Here we show an original method to meet the measurement requirements for ultra-wide bandwidth, ultra-high resolution, and ultra-large dynamic range simultaneously, based on an asymmetric optical probe signal generator (ASG) and receiver (ASR). The ASG and ASR remove the measurement errors introduced by the modulation nonlinearity and enable an ultra-large dynamic range. Thanks to the wavelength-independence of the ASG and ASR, the measurement range can increase by 2 *N* times by applying an *N*-tone optical frequency comb without complicated operation. In an experiment, OVA with a resolution of 334 Hz (2.67 attometer in the 1550-nm band), a dynamic range of > 90 dB and a measurement range of 1.075 THz is demonstrated.

## Abstract (web summary)

Typical methods for optical vector analysis have tradeoffs among resolution, dynamic range, and bandwidth. The authors use an asymmetric optical probe signal generator and receiver to perform attometer resolution measurement over a THz of bandwidth while maintaining high dynamic range, aiming to characterize emerging optical devices.

## Introduction

Recently, optical devices to manipulate the magnitude and phase of optical signals with ultra-high resolution, ultra-wide bandwidth, and ultra-large dynamic range are of fundamental importance for non-Hermitian photonics based on parity-time (PT) symmetry^1^, optical nanoparticle detection^2^, electromagnetically induced transparency^3^, on-chip optical signal processing^4^, ultra-sensitive optical sensing^5^, and so on. These emerging optical devices put forward very urgent and stringent requirements of the measurement technology in terms of resolution, bandwidth, and dynamic range. For example, an ultra-narrow-band fiber Bragg grating with a 3-MHz linewidth^6^, an on-chip optical isolator with a bandwidth of 0.61 MHz^4^, and a high-Q optical micro-resonator with a Q value of 1.7 × 10^10^ (11.4-kHz or 91.2-attometer bandwidth in the 1550-nm band)^7^ were proposed to improve the sensitivity of optical sensing system, which needs an ultra-high resolution optical measurement method to implement the sensing demodulation. Similarly, ultra-narrow bandwidth phenomena, such as spectral hole burning with a 172-kHz notch in Pr:YSO^8^, PT-symmetry breaking with MHz-bandwidth resonance in whispering-gallery-mode microcavities^1^, and ringing phenomenon in chaotic microcavity^5^, has the capability of fine spectral manipulation, which also demands a measurement approach with attometer-level resolution. On the other hand, the spectra of a majority of optical devices and phenomena should be measured in a range of hundreds or even thousands of GHz. For instance, to measure the frequency response of the waveguide–ring resonator^9^, the Fano resonance in all-dielectric metasurfaces^10^, the accidental Dirac cone^11^, and the hydrogen cyanide H^13^C^14^N 2ν_3_^12^, the bandwidth of the measurement systems should be on the order of terahertz. Besides, wide bandwidth and high-resolution measurement are simultaneously needed in some systems^13^. Dynamic range is another important parameter to evaluate a measurement method and concerned by numerous applications. For example, a fiber Bragg grating is expected to have a 50-dB extinction ratio, but the measured result is limited to about 35 dB due to the low dynamic range of the measurement apparatus^14^.

Although researchers have been striving to achieve these goals for decades and some optical vector analyzers were previously reported to obtain the magnitude and phase responses^15–32^, few can perform the measurement with simultaneous ultra-high resolution, ultra-large dynamic range, and ultra-wide bandwidth. Table 1 shows a comparison of different kinds of OVA. As can be seen, the OVA based on the interferometry method^15^ can provide a wide measurement range and a large dynamic range, but a poor resolution. Optical channel estimation^16,31,32^ can reach a sub-MHz resolution, but it is vulnerable in the dynamic range and the compromise on resolution and measurement range. The OVA based on optical single-sideband (OSSB) modulation theoretically has the potential of reaching a sub-Hz resolution^17–27^, but the existence of the high-order sidebands will severely degrade the resolution^23^, introduces considerable measurement errors and restricts the dynamic range^22,23^. In addition, the bandwidth of the electro-optical conversion devices or microwave components usually limits the measurement range to tens of GHz.

**Table 1:** Published results of typical optical vector analyzers

Parameter	Interferometry^15^	Optical channel estimation^16^	OSSB-based OVA	Proposed OVA
Resolution	200 MHz	0.732 MHz^31^	23.4 kHz^27^	334 Hz
Measurement range	Several THz	250 GHz^32^	105 GHz^24^	1.075 THz
Dynamic range	60 dB	25 dB^31^	60 dB	>90 dB

In this article, we propose and demonstrate a method to perform optical vector analysis with simultaneous ultra-high resolution, ultra-large dynamic range, and ultra-wide bandwidth. The basic idea is to generate an asymmetric optical probe signal for carrying on the magnitude and phase responses of the ODUT, and to detect the responses without spectral aliasing by an ASR. The use of the ASG and ASR in the OVA yields three prominent benefits. First, the high-order sidebands inevitably generated by the modulation nonlinearity have a neglected influence on the measurement resolution, because the undesirable components introduced by the high-order sidebands have different frequencies with the useful ones in the ASR, removing the primary source of the measurement error in the conventional modulation-based OVAs. Second, thanks to the extraordinarily high-efficiency frequency-shifting of the acoustooptic modulator (AOM) in the ASG, the unwanted residual sideband^28^ is nearly absent, leading to an enormous sideband suppression ratio. By applying an optimal modulation index, the proposed method can achieve an ultra-large dynamic range. Finally, thanks to the wavelength-independence of the ASG and ASR, any comb line from an optical frequency comb (OFC) can be selected as the optical carrier for a wavelength channel. Thus a large measurement range can be realized by stitching several consecutive channels. Besides, the measurement range in each channel is doubled by using the asymmetric optical probe signal, so when applying an *N*-tone OFC the measurement range can boost to 2 *N* times without complicated operation.

## Results

### Principle and experimental setup

**Fig. 1:**
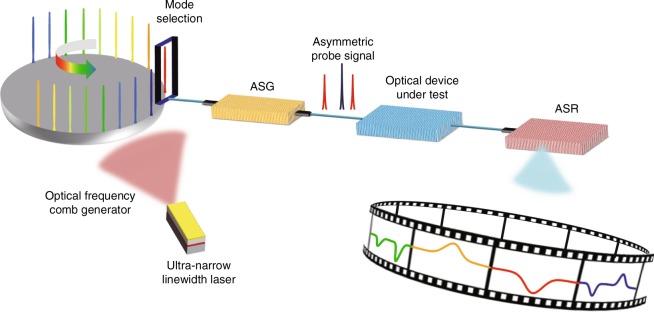
Principle of the optical vector analysis using an optical frequency comb. ASG, asymmetric optical probe signal generator; ASR, asymmetric optical probe signal receiver. The mode-selection module selects a comb line from the OFC signal to generate an asymmetric probe signal by the ASG. After the ODUT, the information carried by the asymmetric probe signal is extracted by the ASR. Changing the wavelength of the mode-selection module, responses in all wavelength channels can be obtained

Figure 1 illustrates the conceptual diagram of the OVA. The optical signal from an ultra-narrow linewidth laser goes through an OFC generator to stimulate an ultra-narrow linewidth OFC signal. A mode-selection module is used to select a comb line, which is sent to an ASG to generate an asymmetric optical probe signal. The asymmetric optical probe signal propagates in the optical device under test and carries on the optical responses in both sides of the comb line. An ASR extracts the spectral response from the asymmetric optical signal, removing the influence of the high-order sidebands and other unwanted components. By sweeping the frequency of the asymmetric optical probe signal via tuning an RF source, the responses in the wavelength channel corresponding to the selected comb line would be obtained. Selecting other comb lines to perform the measurement and stitching the measured responses in all wavelength channels leads to the measurement of wideband responses covered by the OFC (see Methods).

**Fig. 2:**
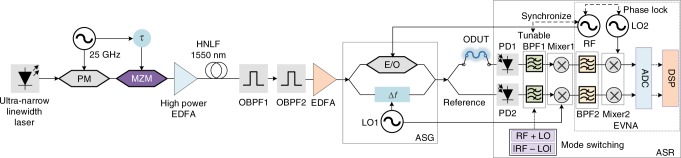
Experimental setup of the proposed OVA. ASG and ASR are used for measurement of magnitude and phase response, and the OFC for measurement range expansion. PM, phase modulator; MZM, Mach-Zehnder modulator; OBPF, optical bandpass filter; EDFA, erbium-doped fiber amplifier; HNLF, highly nonlinear fiber; ASG, asymmetric optical probe signal generator; RF, radio frequency; LO, local oscillator; E/O, electro-optic; ODUT, optical device under test; ASR, asymmetric optical probe signal receiver; PD, photodetector; BPF, optical bandpass filter; ADC, analog-to-digital converter; DSP, digital signal processor

Figure 2 illustrates the experimental setup of the proposed OVA, which consists of an ultra-narrow linewidth laser, an OFC generator, a mode-selection module, an ASG, and an ASR. The laser has a linewidth of ~300 Hz and a long-term frequency stability within ±15 MHz per day. The OFC generator is comprised of a phase modulator, a Mach-Zehnder modulator (MZM), a high power erbium-doped fiber amplifier (EDFA) and a highly nonlinear fiber (1550 nm), which generates a 43-tone OFC with a fixed frequency spacing. Two tunable optical bandpass filters (OBPF) are followed. OBPF1 removes the out-of-band noise, which has a bandwidth of 9 nm, and OBPF2 selects the *n*th (1 ≤ *n* ≤ 43) comb line from the OFC. A second EDFA is inserted to amplify the chosen comb line. In the ASG, the selected comb line is divided into two portions. One portion goes through an AOM, which plays as an effective frequency shifter to have the frequency of the signal upshifted by a frequency of Δ*ω* = 80 MHz. Thus, a frequency-shifted optical carrier is obtained. The other part of the optical signal is modulated by a frequency-sweeping RF signal (denoted as *ω*_e_) at another MZM to generate a carrier-suppressed optical double-sideband (ODSB) modulation signal consisting of two sweeping ±1st-order sidebands. Then, the two signals are combined to form an asymmetric optical probe signal. The asymmetric optical probe signal is further divided into two paths and finally sent to the ASR. The ASR consists of two photodetectors (PDs) and a series of electrical signal processing modules. In the upper path (measurement path), the transmission response of the ODUT modifies the magnitude and phase of the asymmetric optical probe signal. After the square-law detection in PD1, two RF components (denoted as *S*_mea_) carrying the information of the ODUT are generated at the frequencies of |*ω*_e_ − ∆*ω*| and *ω*_e_ + ∆*ω* by beating the ±1st-order sidebands and the frequency-shifted carrier. It is worth noting that the existence of the high-order sidebands does not influence the measurement results due to the difference in frequency except some predictable and removable points where |*ω*_e_ ± Δ*ω*| = *nω*_e_ or |*ω*_e_ ± Δ*ω*| = |*nω*_e_ ± Δ*ω*|. In the lower path (reference path), the asymmetric optical probe signal is directly sent to another PD (PD2 in Fig. 2), such that two reference RF signals (denoted as *S*_ref_) with the frequencies of *ω*_e_ + ∆*ω* and |*ω*_e_ − ∆*ω*| without the phase and magnitude changes introduced by the ODUT are generated. A wideband tunable electronic filter containing a series of electrical switches and parallel tunable bandpass filters (BPFs) is used to select the component of either *ω*_e_ + ∆*ω* or |*ω*_e_ − ∆*ω*|. Mixer1 converts the *ω*_e_ + ∆*ω* and |*ω*_e_ − ∆*ω*| components into *ω*_e_, BPF2 suppresses the unwanted components generated by the Mixer1, and Mixer2 converts the *ω*_e_ component into an intermediate frequency (IF) signal by LO2, which has a fixed frequency difference with the RF signal. The IF receiver would have a larger dynamic range than a baseband receiver because the latter would suffer from quadrature phase errors and power imbalance of IQ-demodulators. To sample the measurement signal and the reference signal, ADCs with large effective number of bits are used. The tuning of BPF1 is synchronized with the adjusting of the RF source, and the RF source, LO1, and the network analyzer are phase-locked to each other. Measurement results are achieved in a digital signal processor (DSP) via *S*_0_ = *S*_mea_ ÷ *S*_ref_ (see Methods), which can also remove the common-mode noise in the measurement and reference paths. To further increase the accuracy of the measurement, the system response and the difference between the measurement and reference paths should be taken into account. To do so, we can remove the ODUT and directly connect the two test ports. In that case, another measured response *S*′_mea_ and its reference *S*′_ref_ will be obtained. We then get a calibration parameter *S*_cal_ = *S*′_mea_ ÷ *S*′_ref_, and an accurate response can be achieved via *S* = *S*_0_ ÷ *S*_cal_. In our implementation, the ADCs, DSP, BPF2, and Mixer2 are realized by a receiver of an electrical vector network analyzer (EVNA), and BPF1 and Mixer1 are implemented by an EVNA extension (Scalar Mixer and Harmonics, R&S ZVA-K4). The incoherent optical structure and coherent electrical receiver make the ASR more sensitive to detect a weak signal and more stable than the interferometry-based method ^15^.

### Implementation of ultra-wide bandwidth

**Fig. 3:**
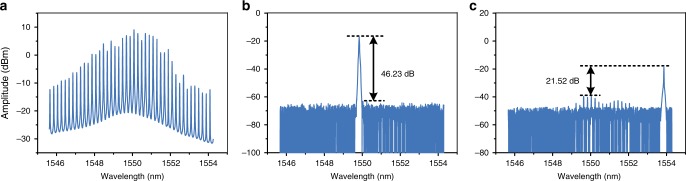
The optical spectra of the OFC and the selected comb line. **a** The generated 43-tone OFC signal measured in a span of more than 8 nm. **b** The comb line in the middle of the OFC selected by OBPF2 which has an SMSR of 46.23 dB. **c** The comb line with an SMSR of 21.52 dB in the marginal area of the OFC selected by OBPF2

An experiment based on the setup shown in Fig. 2 is performed. An OFC signal with a frequency spacing of 25 GHz is generated, and 43 comb lines with relatively high power are selected to be the optical carriers. Figure 3 depicts the optical spectrum of the OFC and the optical carriers selected from the OFC by the mode-selection module. As can be seen in Fig. 3b, c, the side-mode suppression ratio (SMSR) of the selected comb line is 46.23 dB in the middle of the OFC and 21.52 dB in the marginal area. Sweeping the frequency of the RF signal to cover the half of the frequency spacing of the OFC, i.e., 12.5 GHz, the −1st- and +1st-order sideband would probe the full frequency response on both sides of the selected comb line in the range of 25 GHz (one wavelength channel).

**Fig. 4:**
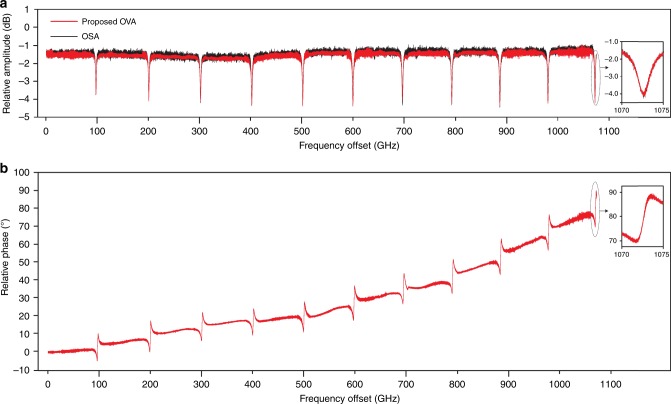
The spectral responses of a H^13^C^14^N gas cell measured by the proposed OVA. **a** The magnitude responses are measured by the proposed method (red line) and an OSA (black line). **b** Since the OSA cannot measure the phase of the optical signal, the phase response can only be obtained by the proposed OVA

Figure 4 shows the frequency responses of a hydrogen cyanide (H^13^C^14^N) gas cell (red lines), which is measured by the proposed OVA. Assume the frequency of the selected comb line is *ω*_o_, the measurement of the wavelength channel is performed by three consecutive segments [*ω*_o_ − 12.5 GHz, *ω*_o_ − 80 MHz], [*ω*_o_ − 80 MHz, *ω*_o_], and [*ω*_o_, *ω*_o_ + 12.5 GHz], corresponding to the beat notes with frequencies of *ω*_e_ − Δ*ω* (*ω*_e_ > Δ*ω*), Δ*ω* − *ω*_e_ (*ω*_e_ < Δ*ω*), and *ω*_e_ + Δ*ω*, respectively. Thanks to the continuity of the responses of the H^13^C^14^N gas cell in any two adjacent channels, the measured responses in different channels can be stitched together regardless of the power difference of the comb lines. In addition, because of the high frequency stability of the OFC, the proposed method can extend the measurement range tremendously, and the measurement resolution will not be deteriorated. In the experiment, all the 43 comb lines with a frequency spacing of 25 GHz are selected one by one as the optical carrier, which boosts the measurement range to 1.075 THz. As a comparison, an optical spectrum analyzer (OSA, hereafter APEX AP2041B) with a resolution of 5 MHz is used to measure the magnitude response of the ODUT, with the result shown as the black line in Fig. 4a. As can be seen, the two measurements agree very well. It should be noted that the signal to noise ratio (SNR) of the measured responses in the marginal areas is lower than that in the middle because the comb lines in those areas have smaller power. The measurement range of the proposed OVA mainly depends on the coverage area of the selected optical comb lines. If the frequency spacing of the OFC and the number of the comb lines are enlarged, the measurement range can be extended to tens or even hundreds of THz in theory. However, according to Beha et al.^33^, the linewidth of the comb lines would be broadened by the phase noise of the RF synthesizer, which may lead to a degraded resolution. It should be noted that in our experiment the linewidth of the RF signal to generate the OFC is at the level of sub-Hz, so the linewidth broadening of the 43 comb lines should be ignorable (<10 Hz).

### Implementation of ultra-high resolution

For the conventional OSSB-based OVA, the existence of the high-order sidebands greatly degrades the wavelength resolution^23^. This resolution limitation factor is fully removed by the proposed method, because the frequencies of the beat notes between the high-order sidebands are *mω*_e_ (*m* = 1, 2,…) and the beating between the high-order sidebands and the frequency-shifted optical carrier generates |*nω*_e_ ± Δ*ω*| (*n* ≥ 2) components, which are different from the required |*ω*_e_ ± Δ*ω*| components and therefore automatically dropped by the coherent electrical receiver in the ASR. The frequency stability of the laser source is another significant limitation for the measurement resolution. By applying the ultra-narrow linewidth laser (OEwaves OE4010) with a short-term frequency stability of 319.5 Hz at 1 ms (see Supplementary Fig. 1), which is measured by an unbalanced Michelson interferometer consisting of a 3 × 3 fiber coupler^34,35^, attometer resolution measurement is possible. With the influence of the high-order sidebands eliminated and a high frequency stability of the laser source, the resolution of the proposed OVA mainly depends on the frequency step of the frequency-swept RF source and the linewidth of the laser. In the experiment, the frequency step of the frequency-swept RF source can reach a few sub-Hz and the laser has a linewidth of 300 Hz, so the proposed method can realize an ultra-high resolution. In addition, the existence of the reference path can remove the common noise or phase jitter between the measurement path and the reference path introduced by the optical source and the environment, so we can eliminate the time-varying measurement errors, ensuring high measurement accuracy, and resolution.

**Fig. 5:**
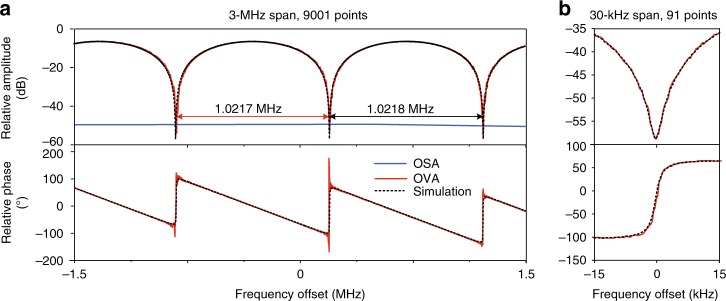
The magnitude and phase responses of a fiber Michelson interferometer. **a** The measurement results in a span of 3 MHz with 9001 points, showing a free spectral range (FSR) of 1.0217 MHz. **b** The measurement results in a span of 3 kHz with 91 points

To verify the ultra-high resolution, a fiber Michelson interferometer with a 100-m arm length difference which has an ideal cosine-type frequency response with a fixed free spectral range (FSR) of 1.0218 MHz is measured by the proposed OVA. Figure 5 shows the measured magnitude and phase responses. In Fig. 5a, the red lines are measured by the proposed OVA, which are composed of 9001 points in the measurement range of 3 MHz, giving a resolution of 334 Hz. The measurements are almost identical to the theoretical simulated responses, which proves the high measurement accuracy of the proposed OVA. The measured FSR is 1.0217 MHz and the calculated arm length difference of the fiber Michelson interferometer is 100.0086 m, which is very close to the designed value. As a comparison, we use the OSA to measure the response of the fiber Michelson interferometer, which has only two points in the 3-MHz range. Figure 5b shows the zoom-in view of the notch in a 30-kHz span. In such narrow bandwidth, the measurement still exhibits a high stability, demonstrating that the proposed method can provide accurate measurement under the high resolution. It should be noted that if the linewidth of the laser can be pushed to several Hz^36^ and the frequency drift is very slow, the resolution of the proposed method might reach Hz-level.

### Implementation of ultra-large dynamic range

**Fig. 6:**
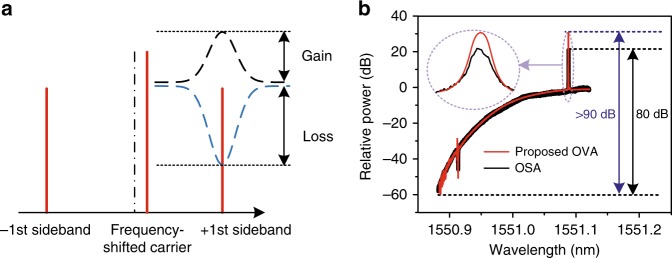
The dynamic range of the proposed OVA. **a** The illustration of the dynamic range. The dynamic range is the difference between the smallest and largest probe signal that can be detected by the ASR. **b** The magnitude response with >90-dB dynamic range measured by the proposed OVA (red line) and an OSA with 80-dB dynamic range (black line)

Many high-Q optical devices not only require ultra-high resolution measurement but also demand that the measurement can provide an ultra-large dynamic range. The maximum dynamic range of the OVA is determined by the ASR. In our implementation, the electrical part of the ASR has a noise floor of −118 dBm and a maximum input power of 22 dBm, and the two PDs (U^2^T 2120RA) have a dark current of 5 nA (−119 dBm) and an output peak voltage of 325 mV (~0 dBm). Thus the power range after the PD is limited to [−118 dBm, 0 dBm] and the maximum dynamic range is restricted to 118 dB. In the conventional modulation-based OVA, the lower bound of the measurement is usually restricted by the residual sideband of the OSSB modulation, the high-order sidebands introduced by the modulation nonlinearity^23,25^ which would submerge the desired signal when the power of the desired signal is less than that of the high-order sidebands, and the residual signals in other unselected channels that can be suppressed to an extremely small value via OBPF2 and the square-law detection in the PD (see methods). For the proposed method, the AOM would not produce mirror sideband, and the high-order sidebands will not affect the measured results according to the above analysis. Therefore, the lower bound of the measurement is mainly determined by the residual signals in other channels, i.e., the SMSR after the mode-selection module. As shown in Fig. 3, the SMSR of the selected carrier is varied from 21 to 46 dB. After square-law detection in the PD, the photocurrents beaten by the ±1^st^ order sidebands and the frequency-shifted carrier are given by $i_{{\mathrm{PD}} \pm 1} = 2\eta \sqrt {P_cP_{ \pm 1}cos((\omega _e \pm \Delta \omega )t + \Delta \varphi )}$, where *η* = 0.65 A/W is the responsivity of the PD, ∆*φ* is the phase difference, and *P*_±1_ and *P*_c_ are the power of the ±1^st^ order sidebands and the frequency-shifted carrier, respectively. The electrical power is *P* = *i*^2^*R*/2 (*i* is the peak current), where *R* = 50 Ω is the load impedance. Thus the power beaten by the ± 1^st^ order sidebands and the frequency-shifted carrier is $P_{{\mathrm{PD}} \pm 1} = \left( {2\eta \sqrt {P_cP_{ \pm 1}} } \right)^2R/2$. Given that both the frequency-shifted carriers and their ±1st order sidebands in the unselected channels are 21–46 dB lower than those in the selected channel, the power of the undesired beating signal would be 42–92 dB smaller than the useful beating signals due to the square-law detection in the PD, which indicates that the proposed method can be used to measure a notch response with a notch depth of 42–92 dB in the selected channel. However, the notch depth of an actual optical device is hard to exceed 70 dB, so we can choose an optical power from the ASG to balance the measurement of loss and gain. Assume the gain and loss only affect the sidebands, we get −118 dBm ≤*P*_PD±1 = _10log_10_$\left( {\left( {2\eta \sqrt {P_cHP_{ \pm 1}} } \right)^2R/2} \right)$ ≤0 dBm and 10log_10_ (*HP*_±1_ + *P*_c_) ≤ 10 dBm (the maximum input power of the PD for linear response is 10 mW, and the other key parameters can be seen in Supplementary Table 1), where *H* is the magnitude response. If the power of the frequency-shifted carrier *P*_c_ is set to 7 dBm and *P*_±1_ = −41 dBm, the proposed OVA can measure a −70-dB notch. In this case, a device with a 48-dB peak gain would amplify the sideband to 7 dBm. Therefore, the maximum dynamic range of the OVA is 118 dB, as shown in Fig. 6a. The channel using the comb line in the marginal area would have a lower dynamic range, since it can merely measure a −42-dB notch and a 48-dB gain peak, reaching a 90 dB dynamic range.

In order to verify the ultra-large dynamic range of the proposed OVA, an experiment is performed with an ODUT consisting of two cascaded programmable optical filters (WaveShaper 4000 s) and an 8-km single-mode fiber (SMF) pumped to provide stimulated Brillouin scattering (SBS) gain. The stop band of the filters is set to be around −60 dB and the 8-km SMF stimulates about 30-dB SBS gain. A comb line in the middle of the OFC is selected as the carrier. Figure 6b shows the magnitude response of the ODUT measured by the proposed OVA (the red line) with a measurement range of 30 GHz. The peak gain provided by the SBS is 31 dB and the notch is more than 59 dB, indicating that the proposed method has a dynamic range of >90 dB. As a comparison, the result (the black line) achieved by the OSA with a resolution of 5 MHz and a dynamic range of around 80 dB is also plotted. The two results agree well in most points except for the peak gain. Due to the higher measurement resolution and larger dynamic range, the proposed OVA can obtain a more accurate measurement. It should be noted that the dynamic range can be further extended if a variable optical attenuator is incorporated before the PD, which dynamically adjust the optical power to the PD.

## Discussion

In the proposed method, the measurement speed is dependent on the number of the measurement points and the IF bandwidth. Smaller IF bandwidth leads to lower noise but results in longer sweep time. Since the approximate measurement time per point can be approximated as 1/*B*_IF_, where *B*_IF_ is the IF bandwidth. Assuming there are *N* channels and *M* points in each channel, the measurement time can be calculated as 1/*B*_IF_ × *M* × *N* + (*N*−1) × 20 ms, where 20 ms is the switching time of WaveShaper 4000 s. The value of the IF bandwidth should be set according to the requirements for noise suppression and measurement speed. Although the proposed method can achieve measurement with simultaneous ultra-wide bandwidth and ultra-high resolution, in practice one would measure an ODUT in a wide bandwidth with a coarse resolution to quickly find the region of interest, and then measure the region of interest using a fine resolution. As a result, in the first step the most important thing is fast and wideband measurement, so the IF bandwidth is usually set to a large value (e.g., 1 MHz to 10 kHz), which provides a fast scan but noisy result. In the second step, high resolution and high accuracy measurement is performed to the region of interest, so the IF bandwidth is usually set to a small value (usually less than 1 kHz), which provides much less noisy results. Using a 10-kHz IF bandwidth, the measurement for total 1.075-THz bandwidth and 1,075,086 points costs about 2 min.

If the ODUT has a birefringence feature, we can measure it by adding a polarization controller before the ODUT and a polarization beam splitter after the ODUT. By adjusting the polarization state to align the fast and slow axes of the polarization beam splitter separately and perform measurement two times, we can obtain the responses in the two orthogonal polarization states. According to the Jones matrix^37^, the polarization response can be calculated.

In conclusion, we have proposed a method to meet the measurement requirements of emerging optical devices for ultra-wide bandwidth, ultra-high resolution, and ultra-large dynamic range simultaneously. The designed ASG and ASR extended the measurement range, removed the majority of measurement errors in the conventional modulation-based OVA, and therefore guaranteed a high resolution and a large dynamic range. By applying an OFC, OVA with a resolution of 334 Hz (attometer level), a dynamic range of 90 dB and a measurement range of 1.075 THz was experimentally demonstrated. The proposed method has the potential to be a prevailing method for characterization of a variety of emerging optical devices and provide support for many frontier types of research, such as on parity-time symmetry, optical nanoparticle detection, electromagnetically induced transparency, on-chip optical signal processing, ultra-sensitive optical sensing, and so on.

## Methods

### Experimental condition

The OFC generator consists of a phase modulator (PM, EOSPACE), a Mach-Zehnder modulator (Fujitsu FTM7938EZ), a high power erbium-doped fiber amplifier (EDFA, Amonics AEDFA-33-B-FA) and a length of highly nonlinear fiber. The frequency responses of the tunable optical bandpass filters (OBPF, WaveShaper 4000 s) are programmable. The high power EDFA (Amonics AEDFA-33-B-FA) excites nonlinear effects to broaden the OFC, and the second EDFA (Amonics AEDFA-35-B-FA) amplifies the chosen comb line. The AOM (Gooch&Housego) produces the frequency-shifted optical carrier and forms the asymmetric optical probe signal together with the MZM. The asymmetric optical probe signal is detected by two PDs (U^2^T 2120RA), and the useful information is extracted by an EVNA (R&S ZVA67).

### Theoretical analysis

Mathematical proof is provided to explain the principle of the proposed OVA. The generated *N*-tone OFC signal writes:

1 $$E_{{\mathrm{OFC}}} = \mathop {\sum}\limits_{m = 1}^N {A_m\delta \left( {\omega - \omega _1 - m\omega _{{\mathrm{rep}}}} \right)} ,$$

where *ω*_1_ is the angular frequency of the first comb line, *ω*_rep_ is the frequency spacing of the OFC, and *A*_*m*_ is the complex amplitude of the *m*th comb line. The selected *n*th comb line can be expressed as:

2 $$\begin{array}{l}E_{{\mathrm{OFC}}}^\prime = {\!} A_n\delta \left( {\omega - \omega _1 - n\omega _{{\mathrm{rep}}}} \right)\hfill \\ \hskip 30pt + \mathop{\sum}\limits_{{\mathop{\scriptscriptstyle{m = 1}}\limits_{m\neq n}}} {\alpha _mA_m\delta \left( {\omega - \omega _1 - m\omega _{{\mathrm{rep}}}} \right)} ,0 {\,} < {\,} \alpha _m {\,} < {\,} 1,\end{array}$$

where *α*_*m*_ is the attenuation of the OBPF applied to the *m*th comb line, which is assumed to be a constant.

The output signal after the AOM is frequency-upshifted by ∆*ω*:

3 $$\begin{array}{l}E_{{\mathrm{AOM}}}\left( \omega \right) = bA_n\delta \left[ {\omega - \left( {\omega _1 + n\omega _{{\mathrm{rep}}} + \Delta \omega } \right)} \right]\hfill \\ \hskip 50pt + b \mathop{\sum}\limits_{{\mathop{\scriptscriptstyle{m = 1}}\limits_{m\neq n}}} {\alpha _mA_m \delta \left[ {\omega - \left( {\omega _1 + m\omega _{{\mathrm{rep}}} + \Delta \omega } \right)} \right]} ,\end{array}$$

where *b* is the conversion efficiency of the AOM.

The other part of the optical signal is modulated by a sweeping RF signal *ω*_e_ at an MZM to generate a carrier-suppressed ODSB signal, which includes the sweeping ±1st-order sidebands and the unwanted components such as the residual carrier, the high-order sidebands, and the components in other channels. After the MZM, the output signal can be expressed as

4 $$\begin{array}{c}E_{{\mathrm{MZM}}}\left( \omega \right) = a_{ + 1}A_n\delta \left[ {\omega - \left( {\omega _1 + n\omega _{{\mathrm{rep}}} + \omega _{\mathrm{e}}} \right)} \right]\\ {\hskip 55pt}+ a_{ - 1}A_n\delta \left[ {\omega - \left( {\omega _1 + n\omega _{{\mathrm{rep}}} - \omega _{\mathrm{e}}} \right)} \right]\\ {\hskip 25pt}+ a_0A_n\delta \left[ {\omega - \left( {\omega _1 + n\omega _{{\mathrm{rep}}}} \right)} \right]\\ {\hskip 92pt} {\hskip -25pt} + \mathop{\sum}\limits_{{\mathop{\scriptscriptstyle{i = - \infty }}\limits_{i \ne \pm 1,0}}}^\infty {a_iA_n\delta \left[ {\omega - \left( {\omega _1 + n\omega _{{\mathrm{rep}}} + i\omega _{\mathrm{e}}} \right)} \right]} \\ {\hskip 100pt}+ \mathop{\sum}\limits_{{\mathop{\scriptscriptstyle{m = 1}}\limits_{m\neq n}}}^N {\mathop {\sum}\limits_{j = - \infty }^\infty {\alpha _ma_jA_m\delta \left[ {\omega - \left( {\omega _1 + m\omega _{{\mathrm{rep}}} + j\omega _{\mathrm{e}}} \right)} \right]} } ,\end{array}$$

where *a*_±1_ and *a*_0_ are the relative amplitudes of the sweeping ±1st order sidebands and the residual carrier, respectively. The asymmetric optical probe signal is generated by combining Eqs. (3) and (4)

5 $$\begin{array}{c}E_{{\mathrm{Probe}}}\left( \omega \right) =bA_n\delta \left[ {\omega - \left( {\omega _1 + n\omega _{{\mathrm{rep}}} + \Delta \omega } \right)} \right] \\ + {\,}b \mathop{\sum}\limits_{{\mathop{\scriptscriptstyle{m = 1}}\limits_{m\neq n}}}^N {\alpha _mA_m\delta \left[ {\omega - \left( {\omega _1 + m\omega _{{\mathrm{rep}}} + \Delta \omega } \right)} \right]} \hfill \\ a_{ + 1}A_n\delta \left[ {\omega - \left( {\omega _1 + n\omega _{{\mathrm{rep}}} + \omega _{\mathrm{e}}} \right)} \right]\hfill \\ +{\,} a_{ - 1}A_n\delta \left[ {\omega - \left( {\omega _1 + n\omega _{{\mathrm{rep}}} - \omega _{\mathrm{e}}} \right)} \right]\hfill \\ +{\,} a_0A_n\delta \left[ {\omega - \left( {\omega _1 + n\omega _{{\mathrm{rep}}}} \right)} \right] \cdot \hfill \\ + \mathop{\sum}\limits_{{\mathop{\scriptscriptstyle{i = - \infty }}\limits_{i \ne \pm 1,0}}}^\infty {a_iA_n\delta \left[ {\omega - \left( {\omega _1 + n\omega _{{\mathrm{rep}}} + i\omega _{\mathrm{e}}} \right)} \right]} \hfill \\ + \mathop{\sum}\limits_{{\mathop{\scriptscriptstyle{m = 1}}\limits_{m\neq n}}}^N {\mathop {\sum}\limits_{j = - \infty }^\infty {\alpha _ma_jA_m\delta \left[ {\omega - \left( {\omega _1 + m\omega _{{\mathrm{rep}}} + j\omega _{\mathrm{e}}} \right)} \right]} } \end{array}$$

Then, the asymmetric optical probe signal enters the measurement path and the reference path. In the measurement path, the asymmetric optical probe signal goes through the ODUT and carries on the magnitude and phase responses of the ODUT. The output signal can be written as:

6 $$\begin{array}{c}E_{{\mathrm{mea}}}\left( \omega \right) {\!} = E_{{\mathrm{Probe}}}\left( \omega \right) \cdot H\left( \omega \right)\hfill \\ = bA_nH\left( {\omega _1 + n\omega _{{\mathrm{rep}}} + \Delta \omega } \right)\delta \left[ {\omega - \left( {\omega _1 + n\omega _{{\mathrm{rep}}} + \Delta \omega } \right)} \right] \hfill \\ + {\,} b \mathop{\sum}\limits_{{\mathop{\scriptscriptstyle{m = 1}}\limits_{m\neq n}}}^N {\alpha _mA_mH\left( {\omega _1 + m\omega _{{\mathrm{rep}}} + \Delta \omega } \right)\delta \left[ {\omega - \left( {\omega _1 + m\omega _{{\mathrm{rep}}} + \Delta \omega } \right)} \right]} \hfill \\ \quad a_{ + 1}A_nH\left( {\omega _1 + n\omega _{{\mathrm{rep}}} + \omega _{\mathrm{e}}} \right)\delta \left[ {\omega - \left( {\omega _1 + n\omega _{{\mathrm{rep}}} + \omega _{\mathrm{e}}} \right)} \right]\hfill \\ \quad +{\,} a_{ - 1}A_nH\left( {\omega _1 + n\omega _{{\mathrm{rep}}} - \omega _{\mathrm{e}}} \right)\delta \left[ {\omega - \left( {\omega _1 + n\omega _{{\mathrm{rep}}} - \omega _{\mathrm{e}}} \right)} \right]\hfill \\ \quad +{\,} a_0A_nH\left( {\omega _1 + n\omega _{{\mathrm{rep}}}} \right)\delta \left[ {\omega - \left( {\omega _1 + n\omega _{{\mathrm{rep}}}} \right)} \right]\hfill \\ \quad + \mathop{\sum}\limits_{{\mathop{\scriptscriptstyle{i = - \infty }}\limits_{i \ne \pm 1,0}}}^\infty {a_iA_nH\left( {\omega _1 + n\omega _{{\mathrm{rep}}} + i\omega _{\mathrm{e}}} \right)\delta \left[ {\omega - \left( {\omega _1 + n\omega _{{\mathrm{rep}}} + i\omega _{\mathrm{e}}} \right)} \right]} \hfill \\ \quad + \mathop{\sum}\limits_{{\mathop{\scriptscriptstyle{m = 1}}\limits_{m\neq n}}}^N {\mathop {\sum}\limits_{j = - \infty }^\infty {\alpha _ma_jA_mH\left( {\omega _1 + m\omega _{{\mathrm{rep}}} + j\omega _{\mathrm{e}}} \right)\delta \left[ {\omega - \left( {\omega _1 + m\omega _{{\mathrm{rep}}} + j\omega _{\mathrm{e}}} \right)} \right]} } ,\end{array}$$

where *H*(*ω*) = *H*_sys_(*ω*)*H*_ODUT_(*ω*), *H*_sys_(*ω*), and *H*_ODUT_(*ω*) denote the transmission functions of the measurement system and the ODUT, respectively.

After square-law detection in a PD (PD1), the generated photocurrent contains components introduced by the sweeping sidebands, the frequency-shifted carrier, the residual carrier, the high-order sidebands, and the components in other wavelength channels. Due to the difference in frequency, the influence by the residual carrier and the high-order sidebands in all wavelength channels is removed. The components generated by the ±1st sidebands and the frequency-shifted carrier can be expressed as

7 $$	i_{{\mathrm{mea, + 1}}}\left( {\omega _{\mathrm{e}} - \Delta \omega } \right) = Ca_{ + 1}A_nbH\left( {\omega _1 + n\omega _{{\mathrm{rep}}} + \omega _{\mathrm{e}}} \right)H^ \ast \left( {\omega _1 + n\omega _{{\mathrm{rep}}} + \Delta \omega } \right)\\ 	\qquad + Ca_{ + 1}b \mathop{\sum}\limits_{{\mathop{\scriptscriptstyle{m = 1}}\limits_{m\neq n}}}^N {\left[ {\alpha _m^2A_mH\left( {\omega _1 + m\omega _{{\mathrm{rep}}} + \omega _{\mathrm{e}}} \right)H^ \ast \left( {\omega _1 + m\omega _{{\mathrm{rep}}} + \Delta \omega } \right)} \right]}, {\mathrm{if }}{\,}\omega _{\mathrm{e}} {\,\,} > {\,\,} \Delta \omega \\ 	i_{{\mathrm{mea}}, + {\mathrm{1}}}\left( {\Delta \omega - \omega _{\mathrm{e}}} \right) = Ca_{ + 1}A_nbH^ \ast \left( {\omega _1 + n\omega _{{\mathrm{rep}}} + \omega _{\mathrm{e}}} \right)H\left( {\omega _1 + n\omega _{{\mathrm{rep}}} + \Delta \omega } \right)\\ 	+ Ca_{ - 1}b \mathop{\sum}\limits_{{\mathop{\scriptscriptstyle{m = 1}}\limits_{m\neq n}}}^N {\left[ {\alpha _m^2A_mH^ \ast \left( {\omega _1 + m\omega _{{\mathrm{rep}}} + \omega _{\mathrm{e}}} \right)H\left( {\omega _1 + m\omega _{{\mathrm{rep}}} + \Delta \omega } \right)} \right]} ,\,{\mathrm{if }}\omega _{\mathrm{e}} {\,\,} < {\,\,} \Delta \omega \\ 	i_{{\mathrm{mea}}, - {\mathrm{1}}}\left( {\omega _{\mathrm{e}} + \Delta \omega } \right) = Ca_{ - 1}A_nbH^ \ast \left( {\omega _1 + n\omega _{{\mathrm{rep}}} - \omega _{\mathrm{e}}} \right)H\left( {\omega _1 + n\omega _{{\mathrm{rep}}} + \Delta \omega } \right)\\ 	+ Ca_{ - 1}b \mathop{\sum}\limits_{{\mathop{\scriptscriptstyle{m = 1}}\limits_{m\neq n}}}^N {\left[ {\alpha _m^2A_mH^ \ast \left( {\omega _1 + m\omega _{{\mathrm{rep}}} - \omega _{\mathrm{e}}} \right)H\left( {\omega _1 + m\omega _{{\mathrm{rep}}} + \Delta \omega } \right)} \right]} ,$$

where *C* is a constant determined by the ±1st sidebands, the frequency-shifted carrier, and the responsiveness of the PD. As can be seen in Eq. (7), the residual sidebands in other channels are suppressed by *α*_*m*_^2^ times.

In the reference path, the other portion of the asymmetric optical probe signal is directly sent to a PD (PD2), generating a photocurrent including the same frequency components with Eq. (7). Without phase and magnitude changes due to the ODUT, we assume that the transmission function *H*_ODUT_(*ω*) = 1 in the reference path. After the progress of *S*_0_ = *S*_mea_ ÷ *S*_ref_, time-varying measurement errors can be eliminated. To remove the time-invariant response *H*_sys_(*ω*) of the measurement system, the two test ports can be directly connected to perform a calibration. In this case, the components with the frequencies of *ω*_e_ + ∆*ω*, |*ω*_e_ − ∆*ω*| can be given by

8 $$	i_{{\mathrm{cal}}, + 1}\left( {\omega _{\mathrm{e}} - {\Delta} \omega } \right) = Ca_{+1}A_{n}bH_{{\mathrm{sys}}}\left( \omega _1 + n\omega _{{\mathrm{rep}}} + \omega _{\mathrm{e}} \right)H_{{\mathrm{sys}}}^ \ast \left( {\omega _1 + n{\omega} _{{\mathrm{rep}}} + {\Delta} \omega } \right)\hfill \\ 	+ Ca_{ + 1}b \mathop{\sum}\limits_{{\mathop{\scriptscriptstyle{m = 1}}\limits_{n\neq n}}} {\left[ {{\alpha}_m^{2} A_{m} H_{{\mathrm{sys}}}\left(\omega_{1} + m {\omega}_{{\mathrm{rep}}} + \omega_{\mathrm{e}} \right) H_{{\mathrm{sys}}}^\ast \left( {\omega_{1} + m {\omega}_{{\mathrm{rep}}} + {\Delta} \omega}\right)}\right]},{\mathrm{if}} {\,\,} \omega_{\mathrm{e}} {\,\,} > {\,\,} {\Delta} \omega \hfill \\ 	 i_{{\mathrm{cal}}, + {\mathrm{1}}}\left( {{\Delta} \omega - \omega _{\mathrm{e}}} \right) = Ca_{ + 1}A_nbH_{{\mathrm{sys}}}^ \ast \left( {\omega _1 + n\omega _{{\mathrm{rep}}} + \omega _{\mathrm{e}}} \right)H_{{\mathrm{sys}}}\left( {\omega _1 + n\omega _{{\mathrm{rep}}} + {\Delta} \omega } \right)\hfill \\ 	+ Ca_{ - 1}b \mathop{\sum}\limits_{{\mathop{\scriptscriptstyle{m = 1}}\limits_{m\neq n}}}^N {\left[ {\alpha _m^{2} A_{m} H_{{\mathrm{sys}}}^\ast \left( {\omega_{1} + m \omega_{{\mathrm{rep}}} + \omega_{\mathrm{e}}} \right) H_{{\mathrm{sys}}}\left( {\omega_{1} + m \omega_{{\mathrm{rep}}} + {\Delta} \omega}\right)}\right]},{\,} {\mathrm{if}} {\,\,} \omega_{\mathrm{e}} {\,\,} < {\,\,} {\Delta} \omega \hfill \\ 	i_{{\mathrm{cal}}, - {\mathrm{1}}}\left( {\omega _{\mathrm{e}} + {\Delta} \omega } \right) = Ca_{ - 1}A_nbH_{{\mathrm{sys}}}^ \ast \left( {\omega _1 + n\omega _{{\mathrm{rep}}} - \omega _{\mathrm{e}}} \right)H_{{\mathrm{sys}}}\left( {\omega _1 + n\omega _{{\mathrm{rep}}} + {\Delta} \omega } \right)\hfill \\ 	+ Ca_{ - 1}b\mathop{\sum}\limits_{{\mathop{\scriptscriptstyle{m = 1}}\limits_{m\neq n}}}^N {\left[ {\alpha _m^2A_mH_{{\mathrm{sys}}}^ \ast \left( {\omega _1 + m\omega _{{\mathrm{rep}}} - \omega _{\mathrm{e}}} \right)H_{{\mathrm{sys}}}\left( {\omega _1 + m\omega _{{\mathrm{rep}}} + {\Delta} \omega } \right)} \right]} \cdot$$

According to the fact that *α*_*m*_^2^∈[−92 dB,−42 dB], *α*_*m*_^2^ is an extremely small value, so the components in other wavelength channels can be ignored. Therefore, the transmission response of the ODUT can be achieved by Eqs. (7) and (8), which can be written as

9 $$\begin{array}{l}H_{{\mathrm{ODUT}}}\left( {\omega _1 + m\omega _{{\mathrm{rep}}} - \omega _{\mathrm{e}}} \right) = \frac{{i_{{\mathrm{mea}}, - {\mathrm{1}}}^ \ast \left( {\omega _{\mathrm{e}} {\,\,} + {\,\,} \Delta \omega } \right)}}{{i_{{\mathrm{cal}}, - {\mathrm{1}}}^ \ast \left( {\omega _{\mathrm{e}} {\,\,} + {\,\,} \Delta \omega } \right)H_{{\mathrm{ODUT}}}^ \ast \left( {\omega _1 {\,\,} + {\,\,} n\omega _{{\mathrm{rep}}} {\,\,} + {\,\,} \Delta \omega } \right)}}\hfill \\ H_{{\mathrm{ODUT}}}\left( {\omega _1 + m\omega _{{\mathrm{rep}}} + \omega _{\mathrm{e}}} \right) = \frac{{i_{{\mathrm{mea}}, + {\mathrm{1}}}^ \ast \left( {\Delta \omega {\,\,} - {\,\,} \omega _{\mathrm{e}}} \right)}}{{i_{{\mathrm{cal}}, + {\mathrm{1}}}^ \ast \left( {\Delta \omega {\,\,} - {\,\,} \omega _{\mathrm{e}}} \right)H_{{\mathrm{ODUT}}}^ \ast \left( {\omega _1 {\,\,} + {\,\,} n\omega _{{\mathrm{rep}}} {\,\,} + {\,\,} \Delta \omega } \right)}},{\mathrm{if}}\,\omega _{\mathrm{e}}{\,\,} < {\,\,} \Delta \omega \hfill \\ H_{{\mathrm{ODUT}}}\left( {\omega _1 + m\omega _{{\mathrm{rep}}} + \omega _{\mathrm{e}}} \right) = \frac{{i_{{\mathrm{mea}}, + {\mathrm{1}}}\left( {\omega _{\mathrm{e}} {\,\,} - {\,\,} \Delta \omega } \right)}}{{i_{{\mathrm{cal}}, + {\mathrm{1}}}\left( {\omega _{\mathrm{e}} {\,\,} - {\,\,} \Delta \omega } \right)H_{{\mathrm{ODUT}}}^ \ast \left( {\omega _1 {\,\,} + {\,\,} n\omega _{{\mathrm{rep}}} {\,\,} + {\,\,} \Delta \omega } \right)}},{\mathrm{if}}\,\omega _{\mathrm{e}} {\,\,} > {\,\,} \Delta \omega ,\end{array}$$

where *H*_ODUT_(*ω*_1_ + *nω*_rep_ + ∆*ω*) is a constant and represents the response at the wavelength of the frequency-shifted carrier. By scanning the RF frequency, the spectral responses of the ODUT *H*_ODUT_(*ω*_1_ + *mω*_rep_−*ω*_e_) and *H*_ODUT_(*ω*_1_ + *mω*_rep_ + *ω*_e_) can be accurately achieved.

## Supplementary information

Supplementary Information

Source Data

## Data Availability

The source data underlying Figs. 3, 3a, b, 5a, b, and 6b are provided as a Source Data file [10.6084/m9.figshare.9892043.v1]. Other data is available from the corresponding author upon reasonable request.
