# Supplementary material for: Optical vector analysis with attometer resolution, 90-dB dynamic range and THz bandwidth
Source: Nat Commun. 2019 Nov 13;10:5135. doi: 10.1038/s41467-019-13129-x (PMC6853945; doi:10.1038/s41467-019-13129-x)
Supplement: Supplementary file 1 — Supplementary Information [file 41467_2019_13129_MOESM1_ESM.pdf]

Supplementary Information

Optical vector analysis with attometer resolution, 90-dB dynamic range and THz bandwidth

Qing et al.

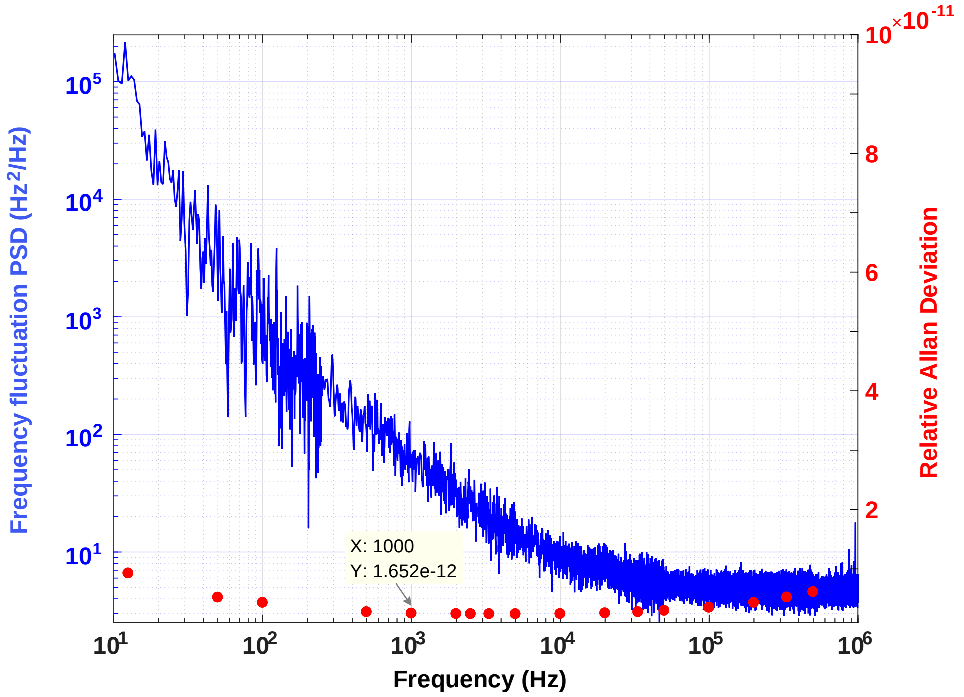

Supplementary Figure 1: Short term frequency stability of the laser source. The frequency noise of the laser source can be described in terms of the power spectral density (PSD), which was measured using an unbalanced Michelson interferometer, and the relative Allan deviation was calculated based on the PSD [1-2]. Frequency fluctuation can be obtained by multiplying the relative Allan deviation by the center frequency of the laser. At the marked point of 10^3 Hz, the relative Allan deviation is 1.652×10^{-12}. Since the center frequency of the laser is 193.4 THz, the frequency fluctuation is 319.5 Hz @ 1 ms.

Supplementary Table 1: Key parameters of the devices and instruments used in the experiment.

Laser source: OE4010[3]		
Instantaneous linewidth	Dynamic linewidth	Frequency stability
300 Hz	300 Hz	±15 MHz per day

Electrical Vector Network Analyzer: R&S ZVA67[4]			
Switching time between channels	Power range of the output port	Trace stability	Dynamic range
< 1ms	− 40 dBm to +18 dBm	0.001 dB	>125 dB

Photodetectors: U²T 2120RA[5]		
Responsivity	Dark current	Maximum input power
0.65 A/W	5 nA	10 mW

Supplementary References

1. Xu, D. et al. Laser phase and frequency noise measurement by Michelson interferometer composed of a 3× 3 optical fiber coupler. *Opt. Express* **23**, 22386-22393 (2015).
2. Zhang, X. et al. Phase-related noise characteristics of 780 nm band single-frequency lasers used in the cold atomic clock. *Chinese Phys. B* **28**, 074209 (2019).
3. https://oewaves.com/hi-q-1-5-micron-lasers-1
4. https://scdn.rohde-schwarz.com/ur/pws/dl_downloads/dl_common_library/dl_brochures_and_datasheets/pdf_1/ZVA_dat-sw_en_5213-5680-22_v1302.pdf
5. https://www.finisar.com/sites/default/files/downloads/hpdv2120r_50_ghz_high-power_photodetector_product_specification_rev_a3.pdf
